# Predictive coding in visual search as revealed by cross-frequency EEG phase synchronization

**DOI:** 10.3389/fpsyg.2015.01655

**Published:** 2015-10-28

**Authors:** Paul Sauseng, Markus Conci, Benedict Wild, Thomas Geyer

**Affiliations:** Department of Psychology, Ludwig-Maximilian-University MunichGermany

**Keywords:** gamma oscillations, memory matching, phase coupling, theta oscillations, visual attention, visual short-term memory

## Introduction

Our experience, memories, and knowledge have modulatory influence on how we perceive the world. Top-down expectancies are supposed to be implemented as templates in our minds. Mental templates are compared against the current sensory input, which can match or mismatch with the aim of minimizing prediction error (Friston, 2005).

But what are the underlying neuronal mechanisms leading to the activation of mental templates and their comparison with sensory input, i.e., predictive coding? Biasing sensory processing by expectancies has been strongly associated with prefrontal brain activity influencing responses in visual cortex (e.g., Summerfield et al., 2006; Olivers et al., 2011; Spaak et al., 2015). Moreover, electrophysiological evidence gathered in patients with prefrontal cortex lesions (Yago et al., 2004) suggests that the prefrontal cortex acts with excitatory drive on extrastriate cortex within three time windows during template matching in visual attention tasks: as early as 100 ms after target onset through selection of spatial locations; during the analysis of non-spatial features of attended objects around 250 ms after target onset; and in a later phase around 300 ms during which discrimination and template matching occur. This recurrent prefrontal drive on higher visual areas can be interpreted as top-down reactivation of target memory traces, thus, the activation of a mental template that needs to be compared to visual input (Desimone and Duncan, 1995).

A common way of analysing interregional transfer of neural signals in the human brain is by means of coherent oscillatory brain activity. Rhythmical brain activity as recorded with the electroencephalogram (EEG) is an indicator for locally highly synchronized neuronal activity. If two distant brain areas are functionally coupled, it is assumed that a higher level of coherent, synchronous neuronal activity can be found between these distant areas than one would expect from chance. Comprehensive work by von Stein and co-workers suggests that long-range interaction between prefrontal and posterior cortices necessary in top-down control of cognitive processes is reflected by neural activity resonating in large-scale networks and therefore oscillating at rather slow frequencies: so called theta and alpha oscillations (von Stein et al., 1999, 2000; von Stein and Sarnthein, 2000). In humans, coherent, synchronous prefrontal to parietal brain oscillatory activity particularly in slower frequency bands (around 5 and 10 Hz) has been observed when a high level of top-down activity is necessary in a range of different visual tasks (see Sauseng and Klimesch, 2008; Sauseng et al., 2010 for reviews). Long-range communication in the monkey brain has also been attributed to rather slow oscillatory activity (in the theta and delta frequency range) whereas it has been suggested that local, fast rhythmical cortical activity (in the gamma frequency band) is associated more strongly with bottom-up visual processing (Bruns and Eckhorn, 2004; Eckhorn et al., 2004; Bastos et al., 2015; Zheng and Colgin, 2015).

## What is the brain oscillatory signature of predictive coding?

There is a large body of evidence that coherent gamma activity in the visual system is associated with binding of visual features in perception (Singer and Gray, 1995; von der Malsburg, 1999; Fries et al., 2007; Singer, 2009 see also Conci et al., 2004), and gamma band activity might be relevant for bottom-up as well as for top-down visual processes (Engel et al., 2001). This idea has been applied to template matching in a theoretical model (memory match and utilization model; MUM) put forward by Herrmann et al. (2004). The MUM suggests two distinct, sequential oscillatory patterns in the brain's response to visual stimuli: The first evoked gamma band response (phase-locked to stimulus onset) at around 100 to 150 ms after stimulus presentation reflects the matching of mental templates to visual input. Next, an induced gamma band response (with jittered phase in relation to stimulus onset) in a time window around 300 ms post-stimulus is related to the usage of this matched information for later, higher cognitive processes. However, the evidence for MUM with regard to memory matching mostly pertains to how semantic knowledge represented in long-term memory affects visual feature binding. An open question is whether there would be a comparable matching process reflected by evoked, phase-locked gamma activity in a visual attention or search task, where a mental template has to be held in *short-term memory*. To our knowledge, so far there is no evidence supporting MUM when information held in short-term or working memory rather than in semantic long-term memory needs to be matched with sensory input.

Sauseng et al. (2008, 2010) put forward a conceptual model which integrates top-down working memory processes as associated by means of fronto-parietal theta activity with bottom-up visual processing manifested by gamma activity in visual areas. They suggested that holding a mental template in mind would increase fronto-parietal phase synchronization (coupling) at theta frequency in time intervals during which a certain visual input is expected (Sarnthein et al., 1998; Sauseng and Klimesch, 2008; Griesmayr et al., 2014). This could reflect the reactivation of a memory trace in working memory, monitored by prefrontal cortex and replayed into higher visual areas. Around 100 ms after onset of visual input (target presentation), posterior theta oscillations would reset their phase. This local resetting of theta phase enables a transient synchronization with high frequency activity in the gamma band range in a time window between 100 and 200 ms post-stimulus. Transient synchronization between theta and gamma phase in posterior parietal cortex has been found to be significantly stronger in trials where expectancies (mental templates) and visual input were matched, i.e., in valid or congruent trials compared to when the template did not match the presented visual target (invalid or incongruent trials; Sauseng et al., 2008; Holz et al., 2010). This suggests that transient parietal phase synchronization between theta and gamma oscillations reflects the integration of top-down controlled mental templates with bottom-up visual processing. The time window around 150 ms post-stimulus for this process to take place is in good agreement with MUM. The major difference, however, is that matching between basic semantic memory and visual input does not require activation of a long-range fronto-parietal theta network. Therefore, there is no necessity for matching incoming visual information with a working memory trace, as reflected by theta activity. Instead, evoked gamma band response as suggested in the MUM would be sufficient for matching semantic knowledge with visual input. However, as soon as a mental template needs to be actively held in working memory, in the alternative model this is achieved via activity of a fronto-parietal theta network; and only the synchronization of theta and gamma at the brain sites where it comes to a spatial overlap between these two oscillations will allow the matching of top-down and bottom-up information.

## Theta:gamma phase coupling—the neural marker of predictive coding?

Recently it has been shown that retention of multiple items in short-term memory and control of working memory functions are also associated with synchronization between theta and gamma oscillations (Canolty et al., 2006; Sauseng et al., 2009; Axmacher et al., 2010; Griesmayr et al., 2010; Kamiñski et al., 2011), and so are processes of context binding in episodic long-term memory (Friese et al., 2013; Staudigl and Hanslmayr, 2013; Köster et al., 2014). This clearly shows that theta:gamma phase coupling is not an exclusive neural marker for predictive coding alone. Cross-frequency coupling plays an important role in a variety of cognitive processes (Jensen and Colgin, 2007; Jirsa and Müller, 2013); and predictive coding can also be reflected by coupling (phase as well as amplitude) of frequencies other than theta and gamma oscillations, or can be associated with a range of other neural processes (Huang and Rao, 2011; Seth et al., 2012; Spaak et al., 2015). Thus, the underlying neural dynamics might strongly depend on the particular cognitive task in question. It seems that for visual search, where a template needs to be held in working memory, theta:gamma coupling is a strong candidate for the neuronal signature of predictive coding, though.

## How to investigate whether evoked gamma activity or theta:gamma phase coupling reflects predictive coding?

Ways of investigating predictive coding are using a visuospatial attentional cueing task or by comparing match and non-match trials in a delayed match-to-sample task. With these paradigms the above described effects of transient theta:gamma phase coupling have been obtained (Sauseng et al., 2008; Holz et al., 2010).

However, predictive coding may derive from both short- and long-term learning in visual search (e.g., Conci et al., 2012 for an overview). One elegant way of how predictive coding can be investigated is the repeated presentation of target-distractor configurations in a visual search task in the so-called *contextual cueing* paradigm (Chun and Jiang, 1998; Geyer et al., 2010a,b; Zellin et al., 2014). The typical finding in contextual cueing is that visual search performance is facilitated in repeated configurations, compared to random target-distractor configurations due to implicit perceptual memory of previously presented search arrays. Geyer et al. (2012) were able to show that memory processes leading to contextual cueing rely on medial temporal lobe (MTL) functions.

Of relevance here is that this form of predictive coding is not so much relying on the matching of semantic long-term information with sensory input as suggested in the MUM. Neither does it require maintenance of an explicit mental template in working memory as suggested in the theoretical framework put forward by Sauseng et al. (2010). Nevertheless, a mental template of target-distractor configurations will be stored in memory, however, without explicit access [and in contrast to paradigms used in (Sauseng et al., 2008) and (Holz et al., 2010) the template will be stored in long-term rather than working memory]. Therefore, what would one expect to be the brain oscillatory correlate of matching such a mental template with visual information in a visual search task? Evoked early gamma activity or rather cross-frequency phase synchronization between theta and gamma activity? To answer this question we first need to discuss brain oscillatory signatures of MTL mnemonic processes.

Theta and gamma activity have both been reported major oscillatory phenomena in the MTL (see Buzsaki, 2006; Lisman and Jensen, 2013; Draguhn et al., 2014). Coherent gamma oscillations have mainly been reported within the MTL (i.e., Fell et al., 2001, 2002). And although there is good evidence of coherent theta activity within the hippocampal formation (see Fell et al., 2003), synchronous theta oscillations between the MTL and the neocortex (particularly prefrontal cortex; Klimesch, 1996; Siapas et al., 2005; Mitchell et al., 2008; Young and McNaughton, 2009) can be found. Consequently, if contextual information stored in the MTL is used in visual search, this information is more likely propagated into the prefrontal cortex via MTL-neocortical theta than gamma networks. Already paced at theta frequency such top-down information could then, as suggested by Sauseng et al. (2010), be transferred to visual areas via fronto-parietal theta networks. Note that context information may be replayed into prefrontal structures because these areas are involved in maintenance of implicit information from MTL memory (e.g., Annac et al., 2013). The main finding of Annac et al. was that implicit contextual cueing vanished when the visual search task was performed concurrently with a secondary spatial working memory task, suggesting that contextual cueing requires working memory resources. In higher visual cortex, top-down predictions would then be matched with bottom-up information by theta:gamma phase synchronization. Following the MUM one might rather expect that bottom-up information is matched with gamma activity already at the level of the MTL. This would make it difficult to impact back on visual cortex in a visual search task, however.

## Conclusion

We conclude that in visual search, predictive coding seems to be reflected by transient coupling of slow oscillations (at theta frequency), alongside more distributed cortical activity, and high frequency oscillatory brain activity occurring particularly in visual areas. Evoked gamma activity as a signature of predictive coding is very compelling in visual perception of semantically congruent information (Herrmann et al., 2004). However, it might not be the most plausible neural correlate of predictive coding in a selective attention task, i.e., in spatial cueing and visual search.

## Author contributions

All four authors were involved in the conceptualization of the topic, the preparation and writing of the manuscript. And all four authors have approved submission of the paper.

## Funding

This research has been supported by the Deutsche Forschungsgemeinschaft DFG (SA 1872/2-1).

### Conflict of interest statement

The authors declare that the research was conducted in the absence of any commercial or financial relationships that could be construed as a potential conflict of interest.
